# Adaptive divergence and the evolution of hybrid trait mismatch in threespine stickleback

**DOI:** 10.1002/evl3.264

**Published:** 2022-01-04

**Authors:** Avneet K. Chhina, Ken A. Thompson, Dolph Schluter

**Affiliations:** ^1^ Department of Zoology & Biodiversity Research Centre University of British Columbia Vancouver BC V6T 1Z4 Canada

**Keywords:** Ecological speciation, hybridization, opposing dominance

## Abstract

Selection against mismatched traits in hybrids is the phenotypic analogue of intrinsic hybrid incompatibilities. Mismatch occurs when hybrids resemble one parent population for some phenotypic traits and the other parent population for other traits, and is caused by dominance in opposing directions or from segregation of alleles in recombinant hybrids. In this study, we used threespine stickleback fish (*Gasterosteus aculeatus* L.) to test the theoretical prediction that trait mismatch in hybrids should increase with the magnitude of phenotypic divergence between parent populations. We measured morphological traits in parents and hybrids in crosses between a marine population representing the ancestral form and twelve freshwater populations that have diverged from this ancestral state to varying degrees according to their environments. We found that trait mismatch was greater in more divergent crosses for both F_1_ and F_2_ hybrids. In the F_1_, the divergence–mismatch relationship was caused by traits having dominance in different directions, whereas it was caused by increasing segregating phenotypic variation in the F_2_. Our results imply that extrinsic hybrid incompatibilities accumulate as phenotypic divergence proceeds.

**Impact Summary**

Researchers interested in speciation aim to identify general processes that cause branching on the tree of life, or speciation. When different species encounter each other and interbreed, they might form hybrids that co‐occur in the parental habitat. If these hybrids fail to persist, then they cannot interbreed with parent lineages and therefore cannot facilitate the exchange of genetic material. This barrier to gene flow promotes speciation. Therefore, it is critical for speciation researchers to understand the factors that affect the fitness of hybrids. It is becoming increasingly appreciated that some hybrids inherit “mismatched” combinations of parental traits, and that this mismatch might render them a poor fit in parental habitats. Our article asks whether the extent of trait mismatches in hybrids increases with the magnitude of adaptive phenotypic differences between parent lineages, which is predicted by theory. We used threespine stickleback fish to test this prediction. Stickleback in freshwater lakes all originated approximately 10,000 years ago from a common marine ancestor. Although the marine ancestor eats zooplankton in the open water, freshwater populations have adapted to a remarkable diversity of habitats—some retain the ancestral zooplanktivorous habitat while others primarily eat large macroinvertebrates in shallow water. We find that hybrids between a marine population and zooplankitivorous freshwater populations have little mismatch, and that the extent of mismatch grows as the freshwater cross parent is increasingly different from the marine. We identify the broad genetic mechanisms that cause this pattern and find that they largely conform to theory. Because mismatch has been linked to reduced fitness in stickleback and other organisms, our study provides new evidence for a potentially general mechanism linking the process of adaptation to the evolution of reproductive isolation

The evolution of reduced hybrid fitness—postzygotic isolation—is a crucial component of the speciation process (Bateson 1909; Dobzhansky 1937; Mayr 1942, 1963; Muller 1942; Coyne and Orr 2004). Postzygotic isolation is often associated with the buildup of intrinsic genetic incompatibilities that accumulate as populations adapt and diverge (Coyne and Orr 1989; Coughlan and Matute 2020). Yet, many young species lack strong intrinsic incompatibilities and hybrid fitness is instead determined by how the phenotype of hybrids facilitates their interactions with the extrinsic ecological environment to influence performance (Grant 1981; Schluter 2000; Nosil 2012). Hybrids can have poor fitness if they have an intermediate phenotype in an environment where there is no intermediate niche (e.g., insects on distinct host plants; Matsubayashi et al. 2010; Bendall et al. 2017; Zhang et al. 2021). Also, the fitness of hybrids can be reduced if they inherit mismatched combinations of traits from parental species (Arnegard et al. 2014; Thompson et al. 2021). Extrinsic selection against hybrids could grow as parent populations diverge if the extent of trait mismatch increases over the course of divergence between populations, in a manner similar to the growth of intrinsic genetic incompatibilities (Coyne and Orr 1989, 1997; Edmands 1999).

Hybrid mismatch occurs in a pair of traits if the hybrid resembles one parent species in one trait and the other parent species in the other trait (Fig. 1A). Dominance can cause mismatch in hybrids if some traits are dominant toward one parent species and other traits are dominant toward the other parent species. A recent synthesis study suggests that mismatch via dominance is common in F_1_ hybrids (Thompson et al. 2021). Segregation variance can also cause mismatch, when individual recombinant hybrids deviate from an intermediate phenotype in opposite ways for different traits (East 1916; Castle 1921; Wright 1931; Schemske and Bradshaw 2002). Because dominance affects the F_1_ more than the F_2_ (Lynch and Walsh 1998), mismatch in F_1_s is primarily expected to be a result of dominance whereas mismatch in recombinant hybrids might be due to one or both dominance and segregation variance.

**Figure 1:**
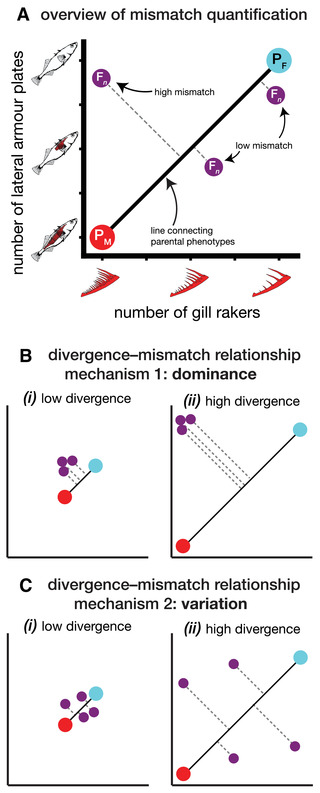
Visual overview of mismatch for two traits. All panels show parent mean phenotypes as large red (marine parent; P_M_) and blue points (freshwater parent; P_F_) and individual hybrids (F_*n*_) as smaller purple points. Panel (A) shows three individual hybrids—one with high mismatch and two with low mismatch. The length of the dashed lines is the “mismatch” quantity. Panels (B) and (C) show the two mechanisms linking mismatch to phenotypic divergence—dominance and variation. Axes and colors are the same as in panel (A) but labels are omitted for clarity. Both divergence–mismatch panels show (i) a case of low phenotypic divergence between parents and (ii) a case of high phenotypic divergence between parents.

Theory predicts that the magnitude of trait mismatch in hybrids should be positively associated with divergence between parent populations. Mismatch can be defined geometrically for a pair of traits as the distance between individual hybrid phenotypes and the line connecting parental mean phenotypes (Fig. 1A). For given dominance coefficients, hybrid mismatch will be low when parent species are phenotypically similar (Fig. 1B[i]). If the parents differ substantially, however, the same dominance will generate a greater magnitude of mismatch (Fig. 1B[ii]). The second reason divergence and mismatch should be associated is that the amount of phenotypic variation in the traits of recombinant hybrids—the segregation variance—is expected to increase with the magnitude of phenotypic divergence between parent populations (Slatkin and Lande 1994; Barton 2001; Chevin et al. 2014) (Fig. 1C). Greater segregation variance results in more extreme mismatched trait combinations appearing in hybrids, and therefore this mechanism is expected to generate greater mismatch as the magnitude of divergence between parents increases.

In this article, we use threespine stickleback fish to test the prediction that trait mismatch in hybrids increases with the magnitude of morphological divergence between parent populations. Freshwater stickleback populations have independently diverged to varying degrees from a common marine ancestor since the last glacial maximum (approx. 10 kya). Contemporary marine populations remain abundant in the sea today and are readily crossed with derived forms. Variation among freshwater populations occurs primarily along a limnetic (i.e., zooplanktivorous) to benthic (i.e., consuming large macroinvertebrates living among the vegetation or lake sediments) axis (Schluter and McPhail 1992; Bell and Foster 1994). Although all have adapted to the freshwater habitat, the more limnetic freshwater populations tend to be phenotypically similar to marine populations whereas the more benthic populations are dissimilar. Because more benthic populations have undergone more phenotypic divergence from the marine ancestor, we hypothesized that their hybrids (in crosses with an extant marine population) will exhibit greater mismatch than those produced from crosses with less divergent populations. To test this hypothesis, we measured morphological traits in hybrids from crosses between the ancestral marine form and 12 derived freshwater populations, quantified mismatch, and investigated its causes.

## Methods

### STUDY SYSTEM

The threespine stickleback is a teleost fish species distributed throughout the coastal areas of the northern hemisphere (Bell and Foster 1994). Stickleback are a longstanding model system for studying the ecological basis of adaptation and speciation (Hagen 1967; McPhail 1969) due in large part to the remarkable phenotypic diversity of populations (Hubbs 1929). Marine stickleback colonized an array of postglacial lakes and have rapidly adapted to prevailing ecological conditions (Schluter 1996). Stickleback that live in lakes containing predators and other competitor fish species (e.g., prickly sculpin) remain similar to the marine population in many morphological traits (Ingram et al. 2012; Miller et al. 2019). By contrast, populations that have evolved in small lakes with few or no predators and competitors often have more derived phenotypes specialized for foraging on large benthic invertebrates. Three lakes (formerly five) contain “species pairs” with reproductively isolated limnetic and benthic populations (McPhail 1984, 1992; Schluter and McPhail 1992)—the limnetics resemble the marine ancestor for many traits, whereas the benthics are among the most derived.

Because adaptive divergence between marine and freshwater populations occurred recently, populations can be readily crossed and typically have few if any “intrinsic” incompatibilities (Hatfield and Schluter 1999; Rogers et al. 2012; Lackey and Boughman 2017). Extant marine populations, within a particular geographic location, are phenotypically similar to the ancestral populations that founded present‐day freshwater populations (Morris et al. 2018). We leveraged this continuum of phenotypic divergence using crosses to test the prediction that hybrid mismatch will be greater when more divergent benthic‐feeding populations are crossed with a marine ancestral population than when this ancestral population is crossed with less divergent zooplanktivorous populations.

We focus specifically on phenotypic divergence as a predictor in our study but previous work in this system allows us to hypothesize about what might be expected if we focused on genetic divergence instead. Studying whole genomes from stickleback populations (single‐species lakes) in coastal British Columbia, Miller (2019) found that mean phenotype (body shape) and was correlated with the major axis of genomic variation among populations, such that populations with similar phenotypes were similar in non‐neutral regions of the genome (i.e., those regions evolving in parallel among independent populations). More zooplanktivorous populations (including Pachena and North lakes) were more similar to the marine populations (including the Little Campbell River) in these genomic regions than were more benthic‐specialized populations (including Bullock and Cranby lakes). Similarly, Jones (2012a) used single nucleotide polymorphism (SNP) data to study genetic variation in the three species pair lakes considered here and the Little Campbell River marine population, as well as in other marine and single‐species lake populations worldwide. The authors found that SNPs under selection were most similar between phenotypically similar populations whereas neutral SNPs largely grouped populations by geography (also see Taylor and McPhail 1999). Wang (2018) replicated these broad pattern using whole genomes. Thus, we would expect estimates of genetic divergence using non‐neutral regions of the genome to parallel our estimates of phenotypic divergence of freshwater populations from the marine form.

### FISH COLLECTION AND HUSBANDRY

**Figure 2:**
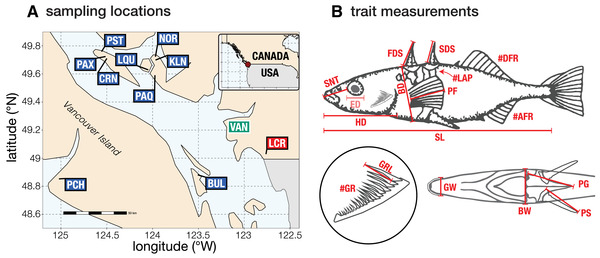
Overview of sampling locations and trait measurements. Panel (A) shows locations where the source populations were collected in British Columbia, Canada. Boxes show collection locations of the marine population (red box; LCR—Little Campbell River) and freshwater populations (blue boxes; left to right: PCH—Pachena Lake; PAX—Paxton Lake; CRN—Cranby Lake; PST—Priest Lake; LQU—Little Quarry Lake; PAQ—Paq (Lily) Lake; NOR—North Lake; KLN—Klein Lake; BUL—Bullock Lake). The green label indicates the location of Vancouver (VAN). Panel (B) shows the measurements of all 16 traits in the dataset and standard length. The upper section of the panel shows the lateral view (traits left to right: SNT—snout length; ED—eye diameter [the transparent shade of red indicates this trait was measured but not analyzed—see “Repeatability” section of methods]; HD—head length; FDS—length of first dorsal spine; BD—body depth; SL—standard length; SDS—length of second dorsal spine; PF—pectoral fin length; #LAP—number of lateral armor plates; #DFR—number of dorsal fin rays; #AFR—number of anal fin rays). The bottom left section of the panel shows a zoomed in drawing of the upper arm of the outer gill raker arch (#GR—number of gill rakers; GRL—length of longest gill raker). The lower right section shows an anteroventral view of the body (GW—gape width; BW—body width; PG—length of pelvic girdle; PS—length of pelvic spine). The upper drawing was originally published by Bell and Foster (1994) and is reused with permission from M. Bell.

Wild fish were collected in British Columbia, Canada, in April–June 2017 and 2018. We sampled twelve freshwater populations from nine lakes (Fig. 2A). Three lakes (Paxton, Priest, and Little Quarry) contain reproductively isolated benthic‐limnetic “species pairs” (McPhail 1992) and thus contributed two populations each). Fish from remaining lakes were single‐species populations presenting a range of intermediate phenotypes from more zooplanktivorous and marine‐like to more benthic. The marine population was collected from the Little Campbell River (Fig. 2A); the population is anadromous—living in the sea and breeding in freshwater, though we refer to them as “marine” here for consistency with previous studies (e.g., Schluter et al. 2021). Wild fish were caught using minnow traps or dip nets. We crossed six gravid marine females with six males from each freshwater population to generate six unique F_1_ hybrid families per population, and also generated four to six nonhybrid (i.e., “pure”) families for each freshwater parental population and the marine ancestor. All offspring were raised in the lab under common conditions (see Supporting Information “Methods”). Crosses were conducted in only one direction (marine as dam) to standardize cytoplasm among hybrid crosses and also because obtaining a sufficient number of wild gravid females for some populations was prohibitively difficult. When lab‐raised fish reached reproductive maturity, F_1_ hybrids from unrelated families were crossed to make three F_2_ families within each cross‐population (with the exception of Paxton Lake benthics which, due to aquarium space constraints in 2018, had only two F_2_ families from the same two F_1_ parent families).

Fish were lethally sampled when individuals in the tank reached a mean standard length of approximately 40 mm. For F_1_s, tanks were subsampled and remaining individuals were raised to produce F_2_s. For F_2_s, entire tanks were lethally sampled. Fish had not reached reproductive maturity at the time of sampling, and we therefore could not determine their sex. Fish were preserved in formalin, stained with alizarin red, and then stored permanently in 40% isopropyl alcohol. See Table S1 and Table S2 for information on sample sizes.

### PHENOTYPE MEASUREMENTS

We measured 16 traits and standard length on stained fish (Fig. 2B). For all traits, we measured at least 100 pure marine parents, and 30 pure freshwater parents, 30 F_1_ hybrids, and 60 F_2_ hybrids from each population and marine‐freshwater cross (all lab‐raised)for trait means, standard deviations (SDs), and sample sizes for all populations). We used a dissecting microscope to count the number of dorsal fin rays, anal fin rays, lateral armor plates, and gill rakers. We also measured the length of the longest gill raker using an ocular micrometer. We photographed the left and ventral sides of each fish with a Nikon D300 camera and used ImageJ (Abramoff et al. 2004) to make linear measurements of body dimensions and bones. Pectoral fins were dissected, immersed in a more concentrated alizarin red stain for at least 24 hours, then photographed. We measured the length of the longest fin ray as pectoral fin length. All measurements with the exception of eye diameter were highly repeatable (*r* ≥ 0.9; see Fig S1), and as a result all traits except eye diameter were used for subsequent analysis. A small number (*n* = 9) of fish had missing second dorsal spines, which caused them to be extreme outliers. We excluded these fish from the analysis. One additional fish that failed to inflate its swim bladder was also excluded.

We size‐corrected all linear measurements by replacing raw measurements with the residuals from simple log‐log (ln‐transformation) linear regressions with standard length conducted across the entire dataset. We controlled for as much of the variation as possible among populations by sampling them at a relatively consistent mean size. Log‐transformation of linear measurements renders trait variances comparable across fish of different sizes (Hatfield 1997). Some measurements are affected if fish are fixed with an open gape, so we further corrected for fixation position by assigning all fish a number (0, 1, or 2) depending on the extent to which the mouth was open and then performing a further correction that removed the effect of fixation position on gape width, snout length, and head length. Trait measurements for missing spines (first dorsal spine or pelvic spine) or pelvic girdle were given a raw value of 0.1 mm before log‐transformation (the log of 0 is undefined). Unlike the second dorsal spine, variation in the presence of these traits is common and does not result in extreme outliers.

Following size‐correction, traits were standardized across the entire dataset to a mean of 0 and an SD of 1. This was done because trait divergence has very different magnitudes for different traits (e.g., 30 plates or 1 mm).

### DATA ANALYSIS

We first investigated whether trait mismatch was associated with the magnitude of phenotypic divergence between parent populations. Following this, we quantify the role of dominance and trait variation in driving this relationship.

#### Software

All data processing and model‐fitting was done using R (R Core Team 2019) using the tidyverse (Wickham 2017). Mixed models were fit using lme4 (Bates et al. 2014) and analysed using lmerTest (Kuznetsova et al. 2014) with the Kenward–Roger approximation for the denominator degrees of freedom (Kenward and Roger 1997). The “map” function in purrr (Henry and Wickham 2019), and associated functions in broom (Robinson et al. 2020), were used to streamline code for iterating models over grouping variables. Partial residuals were plotted using visreg (Breheny and Burchett 2017). for loop code was streamlined with the functions in magicfor (Makiyama 2016). We used the emmeans package (Lenth et al. 2020) and the “cld” function in multcomp (Hothorn et al. 2008) to assist with post hoc comparisons. The functions in the “correlation” package (Makowski et al. 2019) produced correlation matrices.

#### Quantifying phenotypic divergence

We quantified the magnitude of phenotypic divergence between pure marine and freshwater populations as our main predictor of mismatch. To do this, we calculated the multivariate Euclidean distance between each freshwater population's mean phenotype and the marine mean phenotype based on 15 standardized traits. For all estimates of population mean phenotypes we use the unweighted mean of family means, though our conclusions are unchanged if we average across individuals rather than families.

#### Trait mismatch

We quantified trait mismatch as the extent to which individual hybrids deviate from the line connecting parental mean phenotypes (Fig. 1; Thompson et al. 2021). We calculated mismatch between pairs of traits or for all traits together, and because conclusions are broadly similar between the approaches we primarily consider the latter multivariate metric in the main text (see Supporting Information “Results” for pairwise mismatch methods and results). Correlations between pairs of traits in F_2_ hybrids were low (median | *r*_Pearson_ | = 0.2), and most (87%) were not statistically significant at *P* = 0.05, and for this reason we do not use dimensionality‐reduction techniques such as principle components analysis.

Mismatch is the shortest (i.e., perpendicular) Euclidean distance between a hybrid's phenotype and the line that connects the two parental mean phenotypes (Fig. 1). Mismatch (*d*_mm_) was calculated using the standardized traits as:

(1)

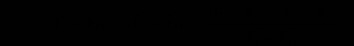

where $\vec{F_{n}},\vec{{\bar{P}}_{M}}$, and $\vec{{\bar{P}}_{F}}$ are the vectors of individual hybrid (F_*n*_ = F_1_ or F_2_), mean marine, and mean freshwater trait values. Individuals from parent populations exhibit deviations from the line connecting parent population means due to their phenotypic variation. The average deviation did not vary among freshwater parent populations (*F*_11, 33.7_ = 1.45; *P* = 0.20) and accounting for parent deviation in our model does not change our conclusions (see archived analysis code).

We tested whether mismatch changed with the magnitude of phenotypic divergence between marine and freshwater parent populations. For simplicity, we analyze mean mismatch values for each cross type within a given marine × freshwater cross, treating population as the replicate. All of our qualitative conclusions are unchanged if we analyze individual‐level data using mixed models with family and population as nested random effects (see archived analysis code). Predictor variables were the (Euclidean) phenotypic divergence between the parental populations, hybrid category (F_1_ or F_2_), and their interaction. Freshwater population was a random effect.

#### Mechanisms underlying the divergence–mismatch relationship

We investigated the causal roles of dominance and phenotypic variation for generating the divergence–mismatch relationship. To determine how dominance affects mismatch for a given marine × freshwater cross, we calculated the mismatch of the mean hybrid phenotype for each population and hybrid generation—hereafter the effect of dominance on mismatch. To determine how phenotypic variation affects mismatch, we subtracted the mismatch of the mean hybrid phenotype (the effect of dominance calculated above) from each individual's unique mismatch value. We took the average of these differences for each family, then averaged these for a single estimate—the effect of variance on mismatch—per population. We used linear models to test whether the effects of dominance and variance on mismatch were associated with the magnitude of phenotypic divergence between parents.

We also quantified general patterns of dominance and phenotypic variation in both F_1_ and F_2_ hybrids to gain intuition about why they drive a divergence–mismatch relationship. We determined whether traits exhibited significant deviations from additivity for each population using linear mixed models where standardized trait values were the response, and the two predictor variables were (i) an additive term (fraction of the genome that is freshwater [P_M_ = 0, F_*n*_ = 0.5; P_F_ = 1]) and (ii) a dominance deviation (fraction of genome heterozygous [P_M_ and P_F_ = 0, F_2_ = 0.5; F_1_ = 1]) (Lynch and Walsh 1998). Family was a random effect. Dominance was only estimated for traits where the marine and freshwater parent populations were statistically distinguishable (*t*‐test *P* < 0.05). To compare dominance among populations, we calculated dominance coefficients by quantifying where the mean (F_1_ or F_2_) hybrid phenotype fell when the marine parent's value was scaled to a value of 0 and the freshwater parent's value was scaled to a value of 1 (transgressive values [<0 and >1] are possible). We tested if dominance coefficients changed with the magnitude of phenotypic divergence between parents using linear models. To evaluate overall patterns of trait variation, we calculated the variance for each trait within each family, then took the average across families. We then fit linear models with mean population variance as the response and both phenotypic divergence between parent populations and hybrid category (and their interaction) as predictors.

## Results

### PATTERNS OF PHENOTYPIC DIVERGENCE AMONG POPULATIONS

Marine × freshwater crosses differed substantially in the magnitude of phenotypic divergence between parent populations (main effect of “population”: *F*_1, 41.8_ = 34.1; *P* < 0.0001; Fig. S2). Freshwater populations were between approximately 3–10 units diverged from the marine, based on 15 standardized traits. The benthic populations from the sympatric species pairs were among the most divergent from the marine ancestor, while two highly zooplanktivorous populations that co‐exist with prickly sculpin were among the least diverged (Pachena Lake and North Lake). The number of traits that differed significantly between the freshwater and marine parents was positively correlated with the magnitude of divergence between parent populations (Fig. S3).

### EVOLUTION OF TRAIT MISMATCH IN HYBRIDS

**Figure 3:**
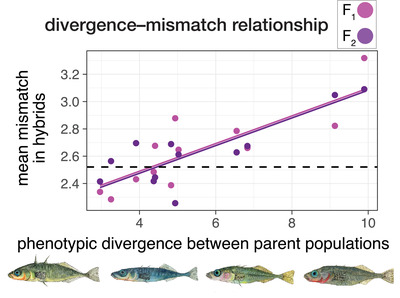
Trait mismatch in hybrids increases with the magnitude of phenotypic divergence between their parents. Each point is the mean mismatch (eq. 1) value across all F_1_ (pink points and lines) or F_2_ (purple points and lines) hybrids for a given marine × freshwater cross (*n* = 12 per hybrid type). The dashed horizontal line shows the mean “mismatch” value (eq. 1) calculated across the freshwater parent (i.e., nonhybrid) populations. Points and regression lines are partial residuals from mixed models. Fish images (by K. Chu) illustrate the range of phneotypes, from left to write: marine form, limnetic, intermediate single‐species form, and benthic.

We found support for the prediction that hybrid trait mismatch increases with the magnitude of phenotypic divergence between parents. Considering all traits together, multivariate mismatch in hybrids was positively associated with the magnitude of phenotypic divergence between parents ($\hat{\beta }$ = 0.10 ± 0.036 [SE], F_1,10_ = 7.71, *P* = 0.020) (Fig. 3). The rate of increase of mismatch with divergence (regression slope) did not differ between F_1_ and F_2_ hybrids (divergence × category interaction *P* = 0.61). Thus, for every unit of multivariate phenotypic divergence between parents, mismatch in hybrids increases by approximately one‐tenth that amount. Figure S4 gives an example of mismatch for two traits where parents have different magnitudes of divergence.

### UNDERLYING CAUSES OF THE DIVERGENCE–MISMATCH RELATIONSHIP

**Figure 4:**
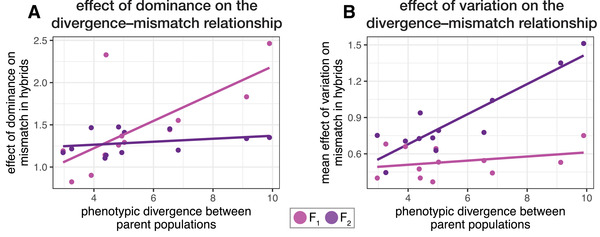
Dominance is the primary cause of the divergence–mismatch relationship in F_1_ hybrids, while variance is the primary cause of the divergence–mismatch relationship in F_2_ hybrids. Panel (A) depicts the mismatch of the mean hybrid phenotype (the *effect of dominance on mismatch*), which is only caused by dominance. The slope is significantly different from zero in F_1_s but not in F_2_s. Panel (B) depicts the mean difference between mismatch values for individual hybrids and the mismatch of the mean hybrid phenotype (the *effect of variance on mismatch*). The slope is significantly different from zero in F_2_s but not in F_1_s. One point is shown per marine × freshwater cross and category (i.e., F_1_ or F_2_; each *n* = 12). Points and regression lines are partial residuals from mixed models.

Dominance was the main cause of the divergence–mismatch relationship in F_1_ hybrids, whereas phenotypic variation was the main cause of this relationship in F_2_ hybrids (Fig. 4).

Mismatch of the mean hybrid phenotype—the effect of dominance on mismatch—increased with the magnitude of phenotypic divergence between parents in F_1_ hybrids ($\hat{\beta }$ [95% CI] = 0.16 [0.052, 0.271]) but not in F_2_s ($\hat{\beta }$ = 0.018 [−0.92, 0.13]; parent divergence × hybrid category interaction term *F*_1,10_ = 5.14, *P* = 0.047) (Fig. 4A). More than three‐fourth (77%) of traits were inherited nonadditively when tested within populations and two‐thirds of traits exhibiting deviations from additivity tended toward recessivity (i.e., F_1_s were more marine‐like). The average dominance of traits differed among traits—for example, most F_1_ hybrids had long heads, similar to the freshwater populations, but also had large pectoral fins, similar to the marine population (Fig. S5). Dominance coefficients typically did not change consistently with the magnitude of phenotypic divergence between parents, although such a pattern was evident for the number of lateral armor plates and the length of pelvic spines (Fig. S6).

We found that the mismatch caused by deviation from the mean hybrid phenotype—the effect of variation on mismatch—increased with the magnitude of phenotypic divergence between parents in F_2_ hybrids ($\hat{\beta }=0.12$ [0.069, 0.18]) but had no effect in F_1_s ($\hat{\beta }$ = 0.017 [−0.039, 0.073]) (Fig. 4B; parent divergence × hybrid category interaction term, *F*_1, 10_ = 3.57, *P* = 0.0051). The relationship between the variance‐effect and divergence was caused by trait variation increasing with phenotypic divergence between parents in F_2_ hybrids but not in F_1_s (Fig. S7). Together, these analyses indicate that the quantitative genetic basis of the divergence–mismatch relationship differs between hybrid generations.

As a result of dominance, mismatch, and divergence between parents itself, the distance of 316 F_1_ and F_2_ hybrids to both the marine and the freshwater parent mean phenotypes—potentially fitness optima—grows with increasing divergence between populations (Fig. S8).

## Discussion

In this study, we used experimental hybridization in stickleback to test whether the extent of trait mismatch in hybrids grows as parent populations diverge phenotypically. Trait mismatch has been associated with reduced individual fitness in recombinant stickleback (Arnegard et al. 2014) and sunflower (Thompson et al. 2021) hybrids, and a growing number of studies have used indirect inference to link mismatch in F_1_ hybrids to reproductive isolation (Vinšálková and Gvoždík 2007; Matsubayashi et al. 2010; Cooper et al. 2018). Studies of fitness landscapes in hybrids (Martin and Wainwright 2013; Keagy et al. 2016) and correlational selection within species (Schluter 1994), also show patterns consistent the hypothesis that selection acts against mismatched trait combinations.

Our study was motivated by the fact that, although previous studies have documented a seemingly general relationship between ecological divergence and barriers to gene flow (Shafer and Wolf 2013), predictions of hypotheses that link adaptive divergence to the evolution of potentially maladaptive hybrid phenotypes remain untested. Comparative studies of speciation (Matute and Cooper 2021) typically study divergence over time, whereas here we consider populations from different lakes that have diverged from a common ancestor to varying degrees in roughly the same amount of time—thus isolating the effect of phenotypic divergence. In support of our prediction, more derived and divergent freshwater parental populations tend to produce hybrids with increasingly mismatched phenotypes when each is crossed to the same marine population representing the ancestral form. The quantitative genetic underpinnings of the divergence–mismatch relationship—dominance in F_1_s and segregation variance in F_2_s—follow from theory and first principles. Below, we discuss these results in the context of speciation research and the genetics of adaptation.

### RELATION TO “INTRINSIC” INCOMPATIBILITIES

Mismatch might be thought of as an ecological and phenotypic analogue to classic “intrinsic” Bateson–Dobzhansky–Muller (BDM; Bateson 1909; Dobzhansky 1937; Muller 1942) hybrid incompatibilities (Coyne and Orr 2004; Arnegard et al. 2014). In the BDM model, populations diverge at multiple loci each of whose transitions is advantageous (or neutral) in the genetic background where fixation occurred. Divergent alleles at loci having separate evolutionary histories might interact negatively when combined in a hybrid, causing reduced fitness. In the phenotypic analogue, populations diverge in multiple traits each of whose changes is advantageous in its own environment and on a given phenotypic background. When combined in a hybrid, (net) dominance relationships and segregation variance might produce mismatch between traits in hybrids, reducing performance and fitness. Such phenotypic incompatibilities imply negative interactions between the underlying genes, as in the BDM model. A phenotypic perspective provides additional insights. Phenotypic incompatibilities are inevitably environment‐dependent. They may be detectable in one environment and not in another (Arnegard et al. 2014). Phenotypic incompatibilities will often be frequency‐ and density‐dependent, at least for traits involved in resource exploitation, implying a more dynamic adaptive landscape for hybrid fitness than in the classic BDM model.

Because mismatch increases with parental divergence, this implies that phenotypic incompatibilities might evolve in a similar “clock‐like” manner to intrinsic post‐zygotic isolation. Coyne and Orr (1989, 1997) were the first to demonstrate that reproductive isolation between populations evolves as a function of divergence time. They found that both premating and intrinsic postzygotic isolation increased with genetic distance (a proxy for time) in *Drosophila*. This work spawned a small industry (Coughlan and Matute 2020; Matute and Cooper 2021) reporting similar patterns in groups as diverse as orchids and fishes (Bolnick and Near 2005; Scopece et al. 2013). In the present study, however, freshwater stickleback populations varying in their degree of divergence from the ancestral marine form are similar in age, all having adapted to varying lake conditions since the end of the last ice age (Wang 2018; Miller et al. 2019). Hence, mismatch is decoupled from neutral genetic divergence and time in this specific case.

The “snowball” model of the accumulation of hybrid incompatibilities, first put forward by Orr (1995), suggests that the number of hybrid incompatibilities should increase faster‐than‐linearly with divergence time. This is because the number of potential pairwise interactions among divergent loci—and thus pairwise incompatibilities—increases at least as fast (“at least” because this does not account for anything above pairwise interactions) as the square of the number of substitutions separating species. Empirical work has found support for this snowball model (Matute et al. 2010; Moyle and Nakazato 2010; Wang et al. 2015). Using the pairwise mismatch data (see Supporting Information), we determined whether each trait pair was significantly mismatched for each cross. In this analysis, we find that the number of trait pairs that are significantly mismatched increases quadratically with the magnitude of phenotypic divergence between parents in F_1_ hybrids (see Fig. S9). In F_2_ hybrids the pattern is significant but linear. We view this analysis as purely heuristic because trait pairs are not independent, though this issue likely also affects other empirical studies of snowballing incompatibilities because the interacting genes are not known (Matute et al. 2010; Moyle and Nakazato 2010). Nevertheless, trait mismatches do seem to snowball in a similar manner to intrinsic incompatibilities (Matute et al. 2010; Moyle and Nakazato 2010). Even though the number of pairs of traits showing mismatch grows faster than linearly with increasing phenotypic divergence between parents, we showed that multivariate mismatch grows linearly with divergence.

### DOMINANCE OF FRESHWATER‐ADAPTIVE TRAIT VALUES

We found that derived traits are typically not dominant in stickleback (see Fig. S5). Although unexpected if adaptation were from new mutation (Haldane 1927), this finding is consistent with what is known about adaptation to freshwater habitats in stickleback proceeding predominantly via the sorting of existing standing genetic variation (Jones et al. 2012; Nelson and Cresko 2018; Roberts Kingman et al., 2021). We also found that deviations from additivity were in a recessive direction (i.e., toward marine) more often than in a dominant direction. These results are consistent with the findings of Miller et al. (2014), who used QTL mapping to measure dominance in an F_2_ marine (from Japan) × freshwater (Paxton Lake benthic) cross, and found that most QTL were additive or partially additive with a slight but significant bias toward recessivity. Finally, with two notable exceptions, dominance coefficients changed idiosyncratically with increasing phenotypic divergence. This finding that dominance is difficult to predict is in agreement with a recent synthesis study that analyzed data from over 100 crosses (Thompson et al. 2021).

Dominance coefficients of two traits—the number of lateral armor plates and the length of the pelvic spine—varied with the magnitude of phenotype divergence between parent populations. Plate number was recessive in crosses between the marine ancestral form and the least divergent freshwater populations and increasingly dominant when the marine was crossed with more derived freshwater populations. Plate reduction in freshwater stickleback populations is known to be largely caused by a large‐effect variant at the *Eda* locus (Colosimo et al. 2005; Archambeault et al. 2020), which is likely to be fixed for the freshwater allele in all but one (North Lake) of the freshwater populations considered here. Previous studies have shown that additional known alleles that reduce plate number in freshwater stickleback populations also modify the dominance of *Eda*'s effect on the number of armor plates (Colosimo et al. 2004). Our result adds to the understanding of this trait by showing that the net contribution of alleles that modify dominance of lateral armor plates depends on the degree of phenotypic divergence. We also found that reduced length of pelvic spines—the freshwater phenotype—was largely dominant in crosses involving less divergent freshwater populations and largely recessive in crosses involving more derived populations. Less is known about dominance modifiers for the length of the pelvic girdle, although the complete loss of the pelvic girdle is governed by recurrent de novo mutation in *PitX1* (Chan et al. 2010). Differences in the direction of the evolution of dominance between these two different armour traits could be associated with their pathways to fixation. The freshwater allele at *Eda* was likely present at the time freshwater populations originated, so dominance modifiers would have fixed afterward. By contrast, the freshwater allele at *PitX1* likely fixed well after substantial reduction of the pelvis had already occurred in some derived populations—this possibility warrants theoretical investigation. Although hypotheses about the evolution of dominance abound (Fisher 1928; Wright 1934), we cannot determine here whether the evolution of dominance documented for these two traits is incidental or adaptive.

### CAVEATS, FUTURE DIRECTIONS, AND CONCLUSIONS

The clearest limitation of our study is its lack of a direct link between mismatch and fitness. In stickleback, selection against mismatched trait combinations has been shown in crosses between freshwater benthic and limnetic populations (Arnegard et al. 2014; Keagy et al. 2016). Selection against natural marine × freshwater hybrids has been inferred in hybrid zones from the steepness of clines (Vines et al. 2016), as well as observations of heterozygote deficit and cytonuclear disequilibrium (Jones et al. 2006). In the Little Campbell River, the source of the marine population used here, Hagen (1967) inferred that selection against marine × freshwater hybrids is “very intense”, although the specific mechanisms of selection were unclear. Clearly, we must begin to conduct comparative studies where mismatch can be linked to fitness directly. In nhybrid zones, biologists can estimate the strength of selection against hybrids by, among other methods, measuring cline width and back‐crossing rates. Future studies could leverage areas of ongoing hybridization to evaluate whether phenotypic mismatch measured in crosses predicts the strength of selection against natural hybrids (Barton and Hewitt 1985; Harrison 1993). Experimental arrays with recombinant hybrids and nonhybrid progenitors could be used to relate mismatch to back‐crossing rates to identify its effectiveness as a barrier to gene flow. Experimental evolution studies could also be used to robustly estimate the generality of the divergence–mismatch relationship and its effect on hybrid fitness. It would be particularly valuable to identify generalities about whether the mismatch–fitness relationship is linear, diminishing, or accelerating. Clearly, more work is necessary to solidify our general understanding of the fitness effects of mismatch.

Some of our findings are more likely to be general than others. In particular, our results regarding F_2_ hybrid phenotypic variation increasing with the magnitude of divergence between parents were predicted from theory (Slatkin and Lande 1994; Barton 2001; Chevin et al. 2014). It is therefore a reasonable expectation that this particular finding might extend to other systems. Because dominance is somewhat idiosyncratic, it is less clear how general our finding that dominance causes a divergence–mismatch relationship in F_1_s will be, though dominance in hybrids is the rule rather than the exception (Thompson et al. 2021). Because the divergence–mismatch relationship we document here might be an important mechanism driving progress toward speciation, establishing which aspects are idiosyncratic and which are general seems worth the effort.

Ultimately, there are many causes of speciation and trait mismatch will be one of many proximate causes of reproductive isolation. Any mismatch would likely operate alongside other well‐documented processes such as assortative mating (Rundle et al. 2000; Coyne and Orr 2004; Jiang et al. 2013) and selection against immigrants (Nosil et al. 2005). Empirical estimates of the relationship between ecological divergence and hybrid fitness (Edmands 1999; Funk et al. 2006; Shafer and Wolf 2013), or neutral divergence and hybrid fitness (Coughlan and Matute 2020; Matute and Cooper 2021), invariably find that these relationships are noisy. Because F_1_ hybrid mismatch is prevalent in many systems (Thompson et al. 2021), it could be an immediate and powerful barrier to gene flow between many diverging lineages. As shown above, it might even “snowball”. Future studies clarifying the importance of mismatch for speciation are sorely needed.

## AUTHOR CONTRIBUTIONS

KAT and DS designed the study. KAT conducted fieldwork, made crosses, and collected samples. KAT and AKC raised animals, collected data, and co‐wrote the first draft of the manuscript. AKC and KAT analyzed the data with input from DS. All authors revised the manuscript.

## DATA ARCHIVING

All data and analysis code underlying this article can be found on Dryad: https://doi.org/10.5061/dryad.2547d7wrp.

Associate Editor: R. Snook

## Supporting information


**Table S1**: Sample sizes that we strove for in this study; see Table S2 for realized sample sizes.
**Table S2**: Sample sizes realized in this study (total *n* fish in study: 1658). Some analyses of individual traits had more individuals than we list here, but individuals needed to have all traits measured to be included in mismatch analyses
**Figure S1**: Repeatability data for all measured traits.
**Figure S2**: Among‐population variation in the phenotypic divergence to the marine ancestor.
**Figure S3**: Phenotypic divergence between parents is positively associated with the number of traits that differ between them.
**Figure S4**: Visualization of pairwise mismatch for two traits using our empirical trait data.
**Figure S5**: Dominance of the freshwater phenotype in hybrids for different traits.
**Figure S6**: Evolution of dominance.
**Figure S7**: Phenotypic variation increases with the magnitude of phenotypic divergence between parents in F2 (purple) hybrids but not in F1 hybrids (pink).
**Figure S8**: The phenotypic distance between hybrids and parent mean phenotype (potentially fitness optima) increases with the magnitude of divergence between parents.
**Figure S9**: The number of mismatched trait pairs ‘snowballs’ with the magnitude of phenotypic divergence between parents, but only in F1 hybrids.
**Figure S10**: Distribution of divergence–mismatch slopes ($\hat{\beta }$) for pairwise analyses.
