# Supplementary material for: Adaptive divergence and the evolution of hybrid trait mismatch in threespine stickleback
Source: Evol Lett. 2022 Jan 4;6(1):34–45. doi: 10.1002/evl3.264 (PMC8802241; doi:10.1002/evl3.264)
Supplement: Supplementary file 1 — Table S1: Sample sizes that we strove for in this study; see Table S2 for realized sample sizes. Table S2: Sample sizes realized in this study (total n fish in study: 1658). Some analyses of individual traits had more individuals than we list here, but individuals needed to have all traits measured to be included in mismatch analyses Figure S1: Repeatability data for all measured traits. Figure S2: Among‐population variation in the phenotypic divergence to the marine ancestor. Figure S3: Phenotypic divergence between parents is positively associated with the number of traits that differ between them. Figure S4: Visualization of pairwise mismatch for two traits using our empirical trait data. Figure S5: Dominance of the freshwater phenotype in hybrids for different traits. Figure S6: Evolution of dominance. Figure S7: Phenotypic variation increases with the magnitude of phenotypic divergence between parents in F2 (purple) hybrids but not in F1 hybrids (pink). Figure S8: The phenotypic distance between hybrids and parent mean phenotype (potentially fitness optima) increases with the magnitude of divergence between parents. Figure S9: The number of mismatched trait pairs ‘snowballs’ with the magnitude of phenotypic divergence between parents, but only in F1 hybrids. Figure S10: Distribution of divergence–mismatch slopes (β^) for pairwise analyses. [file EVL3-6-34-s001.pdf]

Supplementary material

Supplementary methods

Details of crossing protocol and fish husbandry

Female stickleback were selected for spawning when their abdomens were sharply angled at the cloaca and the first egg was visible. We gently squeezed the sides of the female fish's body to release the eggs into a Petri dish containing water from her source habitat (tank, lake, or river water). Mature male stickleback were identified by their bright blue colouration and red throat. Male fish were euthanized with an overdose of MS-222, and then testes were extracted from the body cavity using fine forceps after making a small incision beginning at the cloaca. We used a small paintbrush to release sperm from testes and to ensure that sperm contacted all eggs. The live fish and fertilized clutches were transported to the InSEAS aquatic facility at the University of British Columbia, Vancouver, British Columbia, Canada. All animal care protocols were approved by the Animal Care Committee at the University of British Columbia (application number A16-0044).

All fish were hatched in 100 L aquaria with room temperature between 17 and 19 °C and a photoperiod that followed local dawn and dusk times. Instant Ocean® Sea Salt was added to maintain a salinity of 5 ppt in all tanks. Fry were fed live brine shrimp nauplii. Chopped frozen bloodworms were added to the diet when fish were large enough, and then finally adult-sized fish were fed full size frozen bloodworms and frozen mysis shrimp *ad libitum* (Hikari Bio-Pure®).

We sampled fish for phenotype measurements typically when the mean standard length of a family was approximately 40 mm. Sticklebacks have adult morphology at this stage and are not sexually reproducing. Due to occasional logistical constraints, some tanks were sampled at earlier or later mean standard length sizes. Also, due to logistical constraint, all populations except for Paxton benthic and Paxton limnetic were collected in 2017.

For Review Only

0.0.1 *Repeatability*

We evaluated the repeatability of our measurements to determine the fraction of variance that could be attributable to measurement error. Repeat measurements were made on at least 25 fish. For linear measurements (including pectoral fin length), we took two separate photographs of each fish (or fin) and made the repeated measurements on these separate photographs. Second photographs were made after returning and then removing the fish (or fin) from its storage vial. Count and gill raker measurements were made on the original specimens. In all cases except pectoral fin length, first and second measurements were made more than one year apart.

Data diagnostics

We checked for outliers in the raw data and evaluated outlier individuals to ensure they were not caused by measurement or transcription error. If fish were inadvertently measured twice, we averaged trait values across measurements. Fish with broken second dorsal spines were removed from the dataset. One fish was removed because it had an unusual body shape—qualitatively appearing as if it had failed to inflate its swim bladder—and it was an extreme outlier in Normal Q-Q plots and in standardized residuals vs. leverage plots. Such phenotypes seem to be caused by environmental factors (e.g., a too-powerful air stone) rather than biological factors (e.g., hybrid incompatibilities).

Pairwise trait mismatch

For pairwise mismatch metrics, separate divergence–mismatch regressions were carried out for each pair of traits. We evaluated the statistical significance of the regression models, as well as the distribution of regression coefficients across models to generate inferences about the relationship between adaptive divergence between parents and mismatch in hybrids.

We examined the relationship between phenotypic divergence and hybrid mismatch for pairs of traits at a time. We found that pairwise trait mismatch was significantly associated with parent

For Review Only

trait divergence for 25 of 105 trait pairs in F_1s, and in F_2 hybrids this was 32 of 105 trait pairs. All significant slopes were positive (Fig. S10). The mean absolute slope of significant relationships was approximately 0.08 in both hybrid types. Significant pairwise mismatch regressions typically involved one or both of: pelvic spine length, pelvic girdle length, or lateral plate count, but several divergence–pairwise mismatch regressions lacking those traits were significant (see archived analysis code).

We use the pairwise mismatch data to test the 'snowball' hypothesis (see Discussion). The significance of pairwise mismatch was evaluated using *t*-tests of the null hypothesis that the difference between individual hybrid mismatch and the 'mismatch' of non-hybrid freshwater individuals was 0. *P*-values were Bonferroni-corrected. We summed the number of trait pairs that were significantly mismatched as the basis for the snowball test.

For Review Only

Supplementary table

Table S1: Sample sizes that we strove for in this study; see Table S2 for realized sample sizes.

generation	*n* fam. per pop.	*n* ind. per fam.	total *n* ind. per pop.
P_M	6	16	100
P_F	6	5	30
F_1	6	5	30
F_2	3	20	60

For Review Only

Table S2: Sample sizes realized in this study (total n fish in study: 1658). Some analyses of individual traits had more individuals than we list here, but individuals needed to have all traits measured to be included in mismatch analyses.

population	category	n families	n (total)	mean n per fam.	SD n ind (among fams.)
Little Campbell River (Marine)	P_M	7	103	14.7	11.5
Bullock Lake	P_F	4	30	7.5	4.4
Cranby Lake	P_F	4	40	10	0
Klein Lake	P_F	4	30	7.5	4.4
Little Quarry Lake (Benthic)	P_F	5	28	5.6	3.3
Little Quarry Lake (Limnetic)	P_F	6	30	5	4.9
North Lake	P_F	4	30	7.5	2.1
Paq Lake	P_F	4	30	7.5	4.1
Pachena Lake	P_F	3	30	10	13.1
Priest Lake (Benthic)	P_F	4	31	7.8	2.2
Priest Lake (Limnetic)	P_F	5	30	6	2.6
Paxton Lake (Benthic)	P_F	3	29	9.7	5.5
Paxton Lake (Limnetic)	P_F	5	30	6	3.7
Bullock Lake	F_1	7	32	4.6	1.1
Cranby Lake	F_1	6	32	5.3	0.8
Klein Lake	F_1	6	31	5.2	1
Little Quarry Lake (Benthic)	F_1	6	29	4.8	0.4
Little Quarry Lake (Limnetic)	F_1	6	35	5.8	2
North Lake	F_1	5	30	6	2.2
Paq Lake	F_1	6	30	5	0
Pachena Lake	F_1	6	30	5	0
Priest Lake (Benthic)	F_1	6	35	5.8	2
Priest Lake (Limnetic)	F_1	6	33	5.5	1.2
Paxton Lake (Benthic)	F_1	2	30	15	4.2
Paxton Lake (Limnetic)	F_1	3	39	13	14.7
Bullock Lake	F_2	2	74	37	9.9
Cranby Lake	F_2	3	89	29.7	11.7
Klein Lake	F_2	2	66	33	8.5
Little Quarry Lake (Benthic)	F_2	2	73	36.5	14.8
Little Quarry Lake (Limnetic)	F_2	3	61	20.3	0.6
North Lake	F_2	3	62	20.7	3.1
Paq Lake	F_2	2	61	30.5	0.7
Pachena Lake	F_2	3	60	20	0
Priest Lake (Benthic)	F_2	3	78	26	13
Priest Lake (Limnetic)	F_2	3	59	19.7	1.2
Paxton Lake (Benthic)	F_2	2	59	29.5	10.6
Paxton Lake (Limnetic)	F_2	3	59	19.7	11.2

For Review Only

Supplementary figures

For Review Only

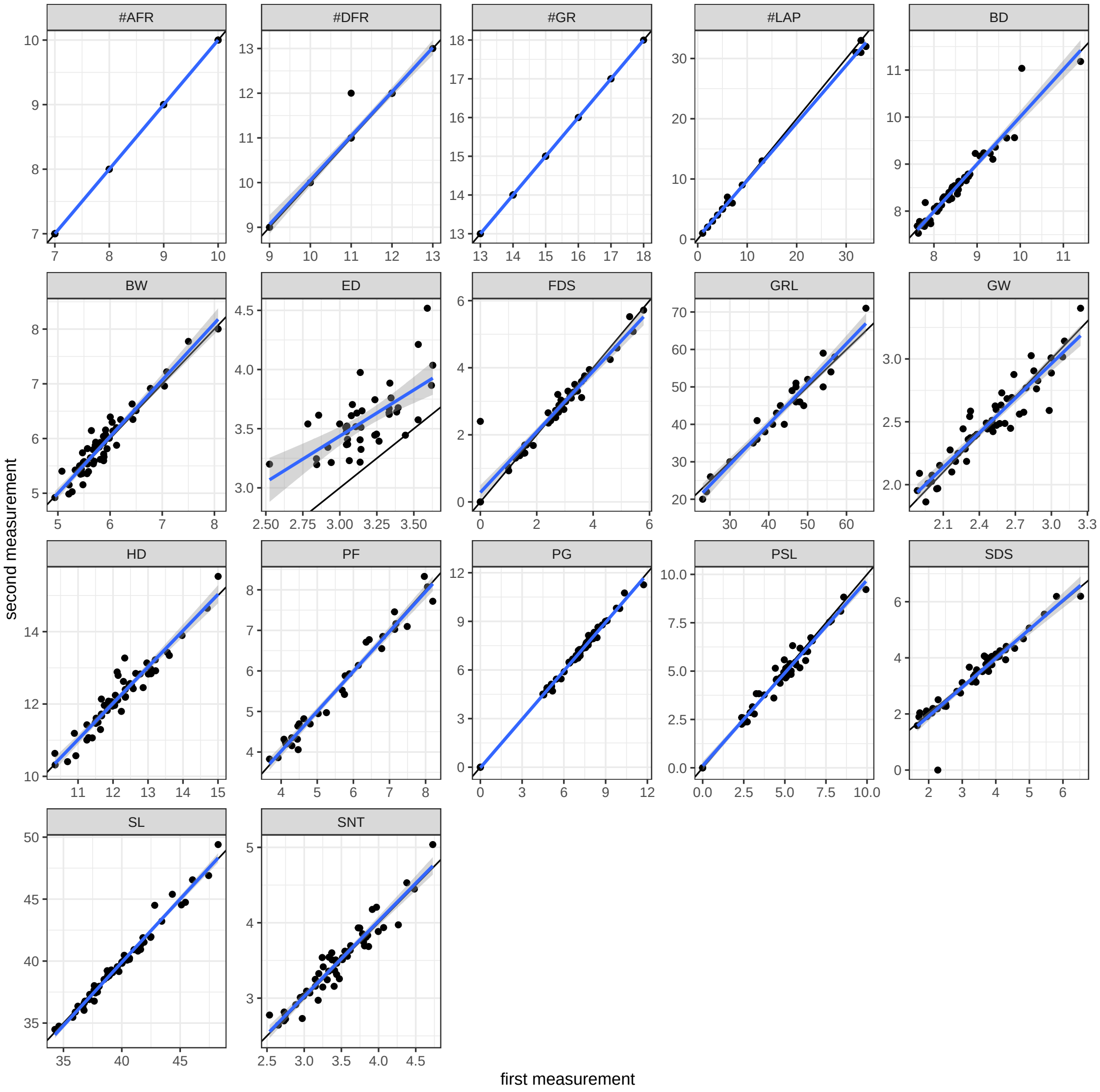

Figure S1: **Repeatability data for all measured traits.** All plots show the first and second measurements made on all traits. Black lines are 1:1 lines, and blue lines are linear regressions. Trait codes are as in Fig. 2B. The fish with a value of '0' for second dorsal spine (SDS) likely had its spine broken off during the time-frame between first and second measurements. All $r_{\text{Pearson}} > 0.9$, except for eye diameter (ED), which was dropped from the analysis.

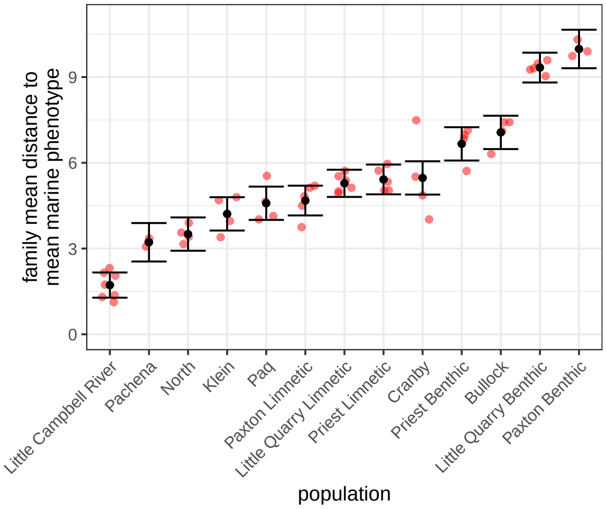

Figure S2: **Among-population variation in the phenotypic divergence to the marine ancestor.** Red points show the phenotypic distance from each family's vector of trait means to the mean marine phenotype. Black points and lines are means and 95 % confidence intervals extracted from the model using `visreg` (Breheny and Burchett, 2017). The Little Campbell River population is not zero because family means are not identical to the mean of family means.

For Review Only

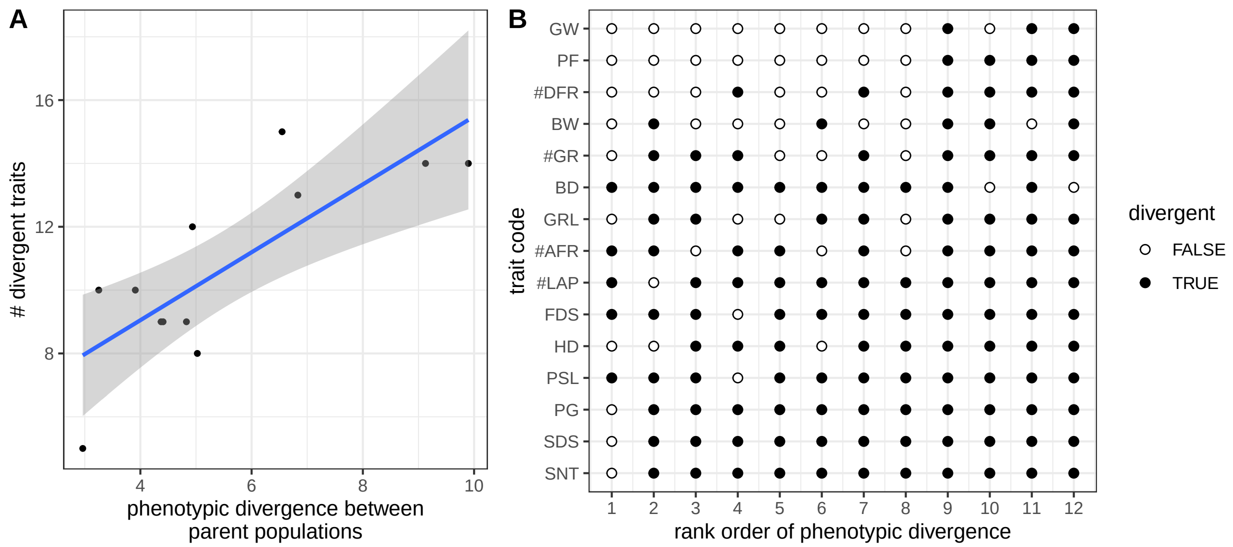

Figure S3: **Phenotypic divergence between parents is positively associated with the number of traits that differ between them.** (A) Each point is one freshwater population. The number of traits is the number that differ significantly between the freshwater population and the marine ancestor according to a Bonferroni-corrected t-test. For every unit of multivariate phenotypic divergence, there is one additional divergent trait ($\hat{\beta}$ = 1.02 $\pm$0.24; $F_{1,10}$ = 18.4; P = 0.0016). Panel (B) shows whether or not individual traits (codes as in Fig. 2B) differ between the freshwater and marine parent for all twelve populations set out on the x-axis by rank-order of phenotypic divergence (see Fig. S2 for rank order).

or Review Only

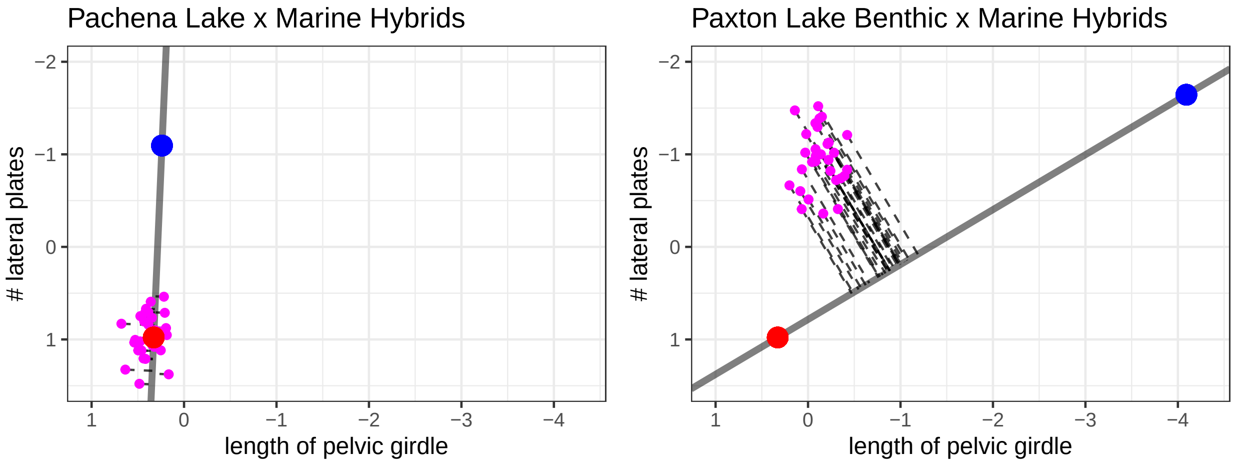

Figure S4: **Visualization of pairwise mismatch for two traits using our empirical trait data.** Each plot shows the scaled phenotype data used in main text analyses. The red points indicate the mean of the freshwater parent population and the blue points indicate the mean of the marine parent population. Mismatch is the length of the dashed (perpendicular) line connecting black points—individual F_1 hybrids—to the line connecting parent mean phenotypes. The higher average mismatch of the Paxton benthic hybrids (μ = 1.54) than Pachena Lake hybrids (μ = 0.087) results from opposing dominance: the pelvic girdle phenotype resembles the marine ancestor whereas the plate number is similar to the freshwater parent.

For Review Only

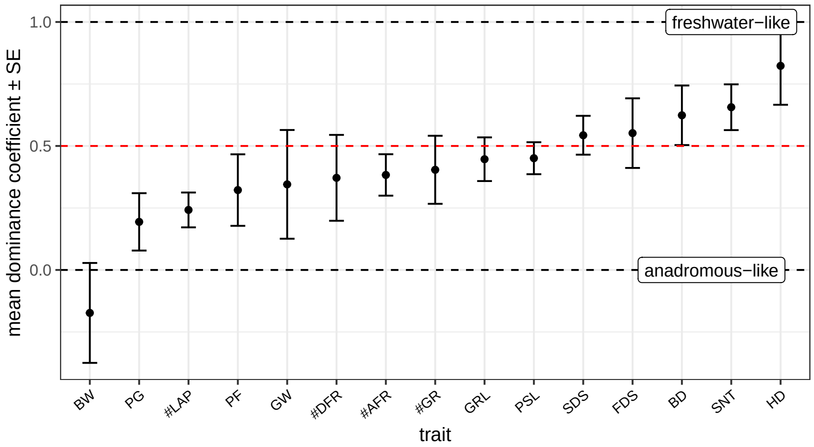

iew Only

Figure S5: **Dominance of the freshwater phenotype in hybrids for different traits.** We plot the mean dominance coefficient—calculated across all populations—for all measured traits (F_1 hybrids only). Complete recessivity (i.e., marine-parent-like) is a value of 0, additivity is a value of 0.5, and complete dominance (i.e., freshwater-parent-like) is a value of 1. Points depict the mean value. The dashed lines at 0 and 1 represent the ancestral marine parent and derived freshwater parent trait values, respectively, and the red dashed line at 0.5 represents the mid-parent value.

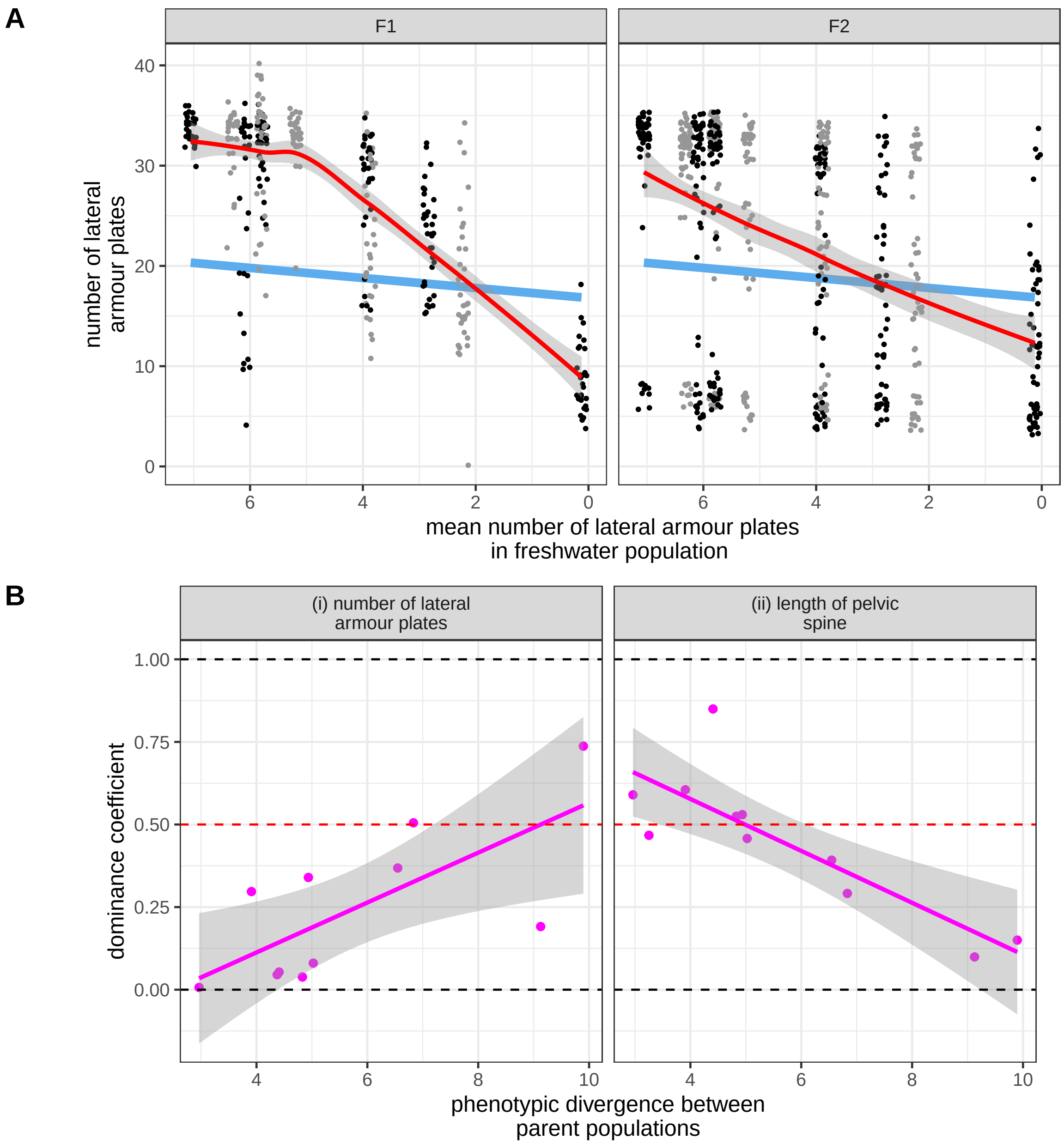

Figure S6: **Evolution of dominance.** Panel (A) shows raw trait data lateral plate counts for both F_1 (left) and F_2 (right) hybrids; F_2s are shown for interest's sake only. The red line is a loess-smooth fit to the data, and the blue line is fit to the parental midpoint. The *x*-axis is reversed and more derived populations are on the right. Grey and black coloured points simply demarcate populations with adjacent mean divergence values (as in chromosomes on a Manhattan plot). As seen in the F_1s, dominance evolves towards less recessivity as divergence proceeds. Panel (B) shows dominance coefficients (as in Fig. S5) for F_1 hybrids for both armour plates (left) and pelvic spine length (right). Panels A and B(i) show the same data plotted in different ways (we do not show the raw data for pelvic spine length since the high variability renders visualization challenging). Pelvic spines are increasingly recessive as divergence proceeds.

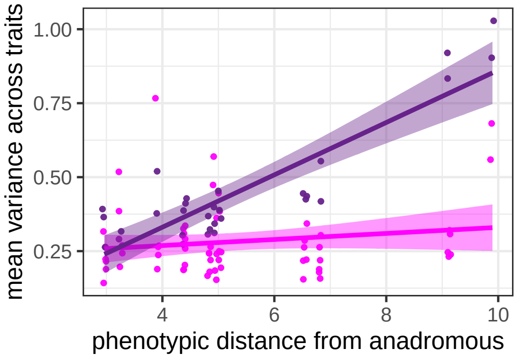

For Review Only

Figure S7: **Phenotypic variation increases with the magnitude of phenotypic divergence between parents in F_2 (purple) hybrids but not in F_1 hybrids (pink).** Points represent the mean of variances across all 15 traits within each independent family (minimum *n* = 5). F_1 and F_2 hybrids are distinguished by colour ($F_{1\text{—pink; }F_2\text{—purple}}$). Linear measurements are ln-transformed, so this result is not simply due to scaling means and variances (count data are raw but values are *lower* in more derived crosses for all meristic traits). Points are horizontally jittered to ease visualization. This graph compliments the 'variance-effect' analysis in the main text by showing patterns of raw trait variance without regard for mismatch.

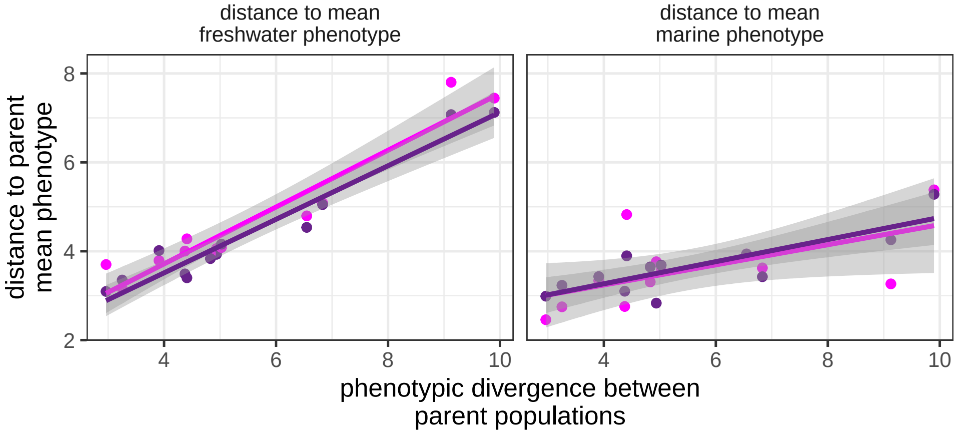

Figure S8: **The phenotypic distance between hybrids and parent mean phenotype (potentially fitness optima) increases with the magnitude of divergence between parents.** Such a pattern would be evident without mismatch and without dominance, so the primary purpose of this figure is to visualize how the effects of dominance lead to differences in the distances to parent means ($F_{1\text{—pink; }F_2\text{—purple}}$). Because traits tend to be recessive (i.e., closer to the marine parent), the slope of the divergence–optima distance relationship is shallower when the marine parent is used as the reference phenotype.

For Review Only

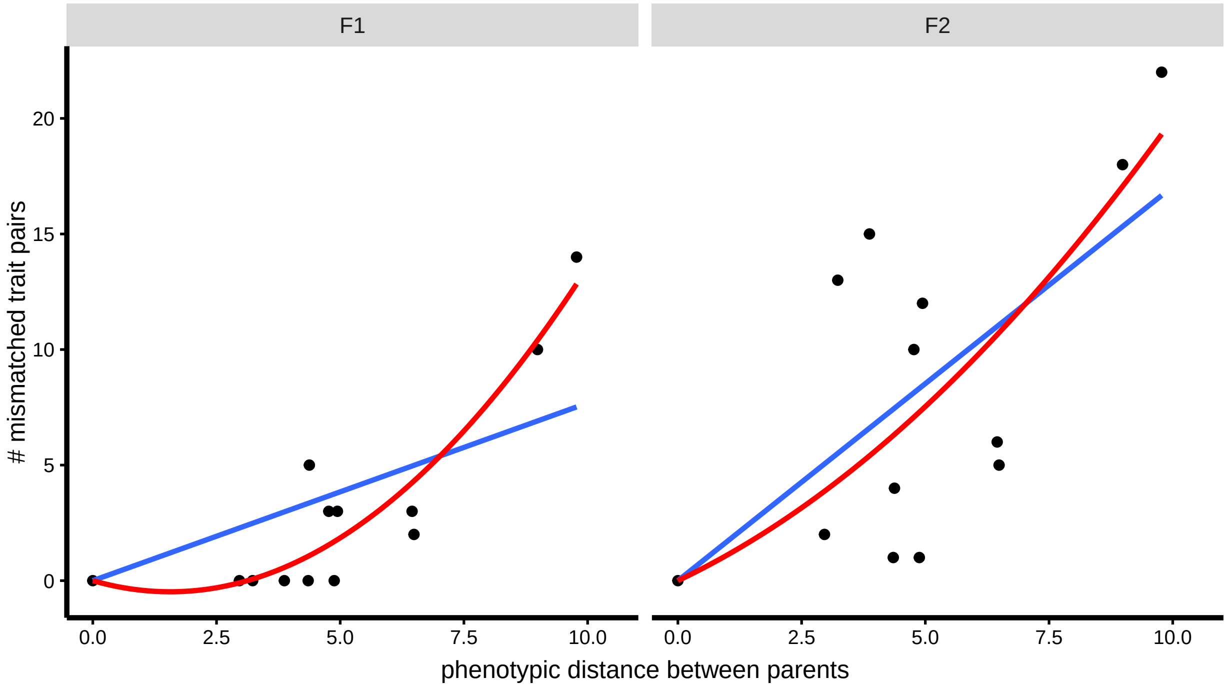

Figure S9: **The number of mismatched trait pairs 'snowballs' with the magnitude of phenotypic divergence between parents, but only in F_1 hybrids.** The y-axis shows the number of trait pairs with significant mismatch (see supplementary text). The plot and regression lines are modelled after the 'snowball' studies of Moyle and Nakazato (2010) and Matute et al. (2010). The blue lines are linear regressions and the red lines are quadratics. Results hold if the intercept is not forced through zero, and if the 'origin' datum is omitted.

review only

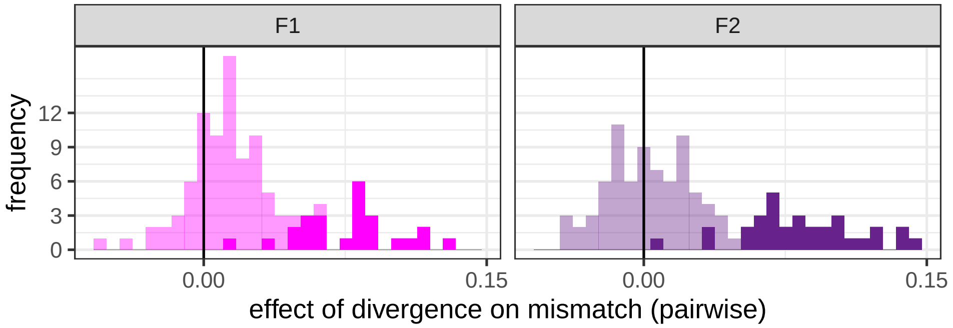

Figure S10: **Distribution of divergence–mismatch slopes ($\hat{\beta}$) for pairwise analyses.** We are showing the frequency distribution of slopes for the pairwise mismatch analyses in F_1 and F_2 hybrids. These slopes capture how mismatch for a pair of traits changes with the magnitude of phenotypic divergence between parents for those two traits. Significant slopes (*) are shown in a darker shade than non-significant (n.s.) slopes.

For Review Only

Data availability statement

The data that support the findings of this study are openly available on Dryad at https://urldefense.com/v3/__https://doi.org/10.5061/dryad.2547d7wrp__;!!N11eV2iwtfs!7Z6R_LDyiQQTTVUeBJKonDhwFf1yTNEXmtEDwhidr_QXCv9G25s0UA7p6ifhOFxSye8A$.

https://urldefense.com/v3/__https://doi.org/10.5061/dryad.2547d7wrp__;!!N11eV2iwtfs!7Z6R_LDyiQQTTVUeBJKonDhwFf1yTNEXmtEDwhidr_QXCv9G25s0UA7p6ifhOFxSye8A$

For Review Only
